# Epidermal cyst protruding from the occipital region of the scalp: a case report

**DOI:** 10.1097/MS9.0000000000001406

**Published:** 2023-10-17

**Authors:** Alok Dahal, Rakesh K. Gupta, Justin J. Malla, Diwakar Koirala, Aashish Baniya

**Affiliations:** Department of Surgery, B.P. Koirala Institute of Health Sciences, Dharan, Nepal

**Keywords:** case report, epidermal cyst, occipital region

## Abstract

**Introduction and importance:**

Epidermal cysts are the most common subcutaneous tumor typically observed on the scalp, face, neck, back, or trunk. Epidermal cysts conventionally range in size from 1 to 5 cm, with sizes greater than 5 cm rarely reported.

**Case presentation:**

Here, we present a case of a 58-year-old female housewife who presented to our surgery outpatient department (OPD) with a history of a mass in the left occipital region. The mass was first noticed 3 years back and was small and associated with mild itching. The mass progressively increased in size over the course of 3 years. Her present complaint was a painful mass associated with itching, headache, and pus discharge when compressed. The pain was relieved by taking medication.

**Clinical discussion:**

The epidermal cyst was confirmed on biopsy, and subsequent excision of the cyst was done under general anesthesia; the occipital bone was eroded by the inferior part of the cyst – reconstruction of scalp defect done by rotational scalp flap.

**Conclusion:**

Epidermal cysts, being a slow-growing benign tumor, can pose diagnostic difficulties, especially when located in the scalp area. In addition to that, when cranial bones and intracranial structures are affected by the cyst, they can even lead to complications and interventional difficulties.

## Introduction

Epidermal cysts are the most common subcutaneous tumor typically seen on the scalp, face, neck, back, or trunk. Epidermal cysts conventionally range from 1 to 5 cm in size, and size greater than 5 cm are rarely reported^1^. Epidermal cysts that are seen commonly are slow-growing tumor. Several pathophysiology applies to epidermal cysts. Sebaceous gland duct obstruction in the hair follicle can result in the long, narrow channel opening in the surface comedones. Additional causes include a sebaceous duct developmental defect or deep epidermal cell implantation brought on by a penetrating injury or prior surgery. Additionally, the genesis of palmoplantar epidermal cysts has been linked to the human papillomavirus. Trauma is considered to be the main cause. However, lesions can present even in the absence of trauma if the trauma has occurred several years back^2^. Atheroma is the term used to describe a cyst that contains keratin and purulent exudates that are formed by bacteria and/or an epidermal lining with a granular layer^3^. In our study, we present a case of localized epidermal cyst in the occipital region of the scalp.

## Case presentation

**Figure:1:**
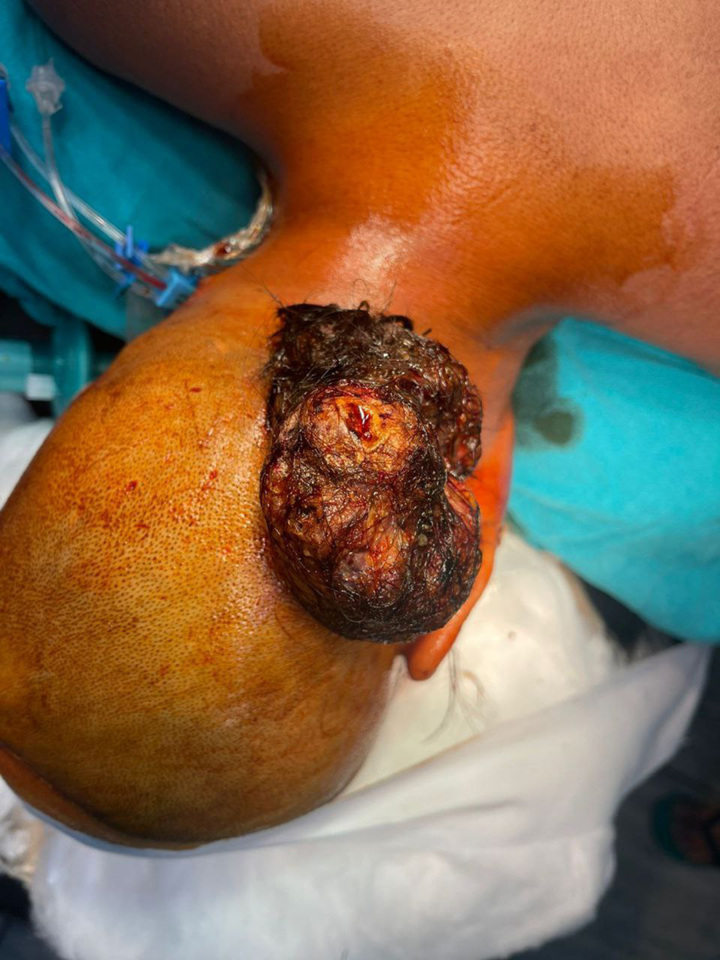
Preoperative figure of the epidermal cyst.

A 58-year-old female housewife presented to our surgery outpatient department (OPD) with a history of a mass in the left occipital region (Fig. 1).

The mass was first noticed 3 years back and was small, associated with mild itching. The mass progressively increased in size over the course of 3 years. Her present complaint was a painful mass associated with itching, headache, and pus discharge when compressed. The pain is relieved by taking medication (non-steroidal anti-inflammatory drugs). There was no history of fever, trauma, vomiting, blurring of vision, neck rigidity, or symptoms of focal neurological deficits. When she presented to the surgery OPD 18 months back, she was diagnosed with hypertension for the first time, for which she has been taking antihypertensives. For the same reason, the excision of the lesion was postponed. There is no history of diabetes mellitus (DM); she is a non-smoker and non-alcoholic. There is no family history of a similar lesion. She has been married for 40 years with Gravida-3 and Parity-3 with a menopausal status of 3 years.

On systemic examination, the respiratory system does not show any abnormal findings. Cardiovascular system auscultation shows normal S1 and S2 with no added sounds of murmur. Abdominal examination was also found to be normal. Central nervous system examination shows no neurological deficits with Glasgow Coma Scale 15/15.

On local examination, the mass was present on the left occipital region over the scalp; it was immobile, soft, rugged surface, well defined, measuring 6 cm×5 cm×7 cm (height×width×length). The overlying skin was slightly protuberant. The adjacent skin was normal (Fig. 1). Her history revealed no previous surgery and she did not recall any previous injury.

On investigations, hemoglobin level was abnormal with 6.4 g/dl. Other complete blood count and differential blood count findings were normal without any deviation – serology of HIV and HBsAg negative. The renal function test and liver function test were all normal. The electrolyte panel was normal. Chest X-ray antero-posterior view was also normal.

**Figure:2:**
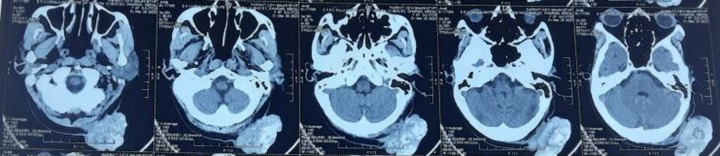
Computed tomography findings.

Computed tomography imaging (Fig. 2) revealed a lobulated heterogeneous lesion with hyperdense content (likely calcification) protruding in the occipital lesion of size: 5.4×4.8×7.3 cm (antero-posterior, transverse, craniocaudal) and lack of other pathological changes within the brain. The tumor was suspected to be a calcified epidermal cyst. The surgery was performed under general anesthesia, and excision of the cyst was done; the occipital bone was eroded by the inferior part of the cyst. Reconstruction of scalp defect was done by rotational scalp flap, and then it was sent for histopathological examination.

**Figure:3:**
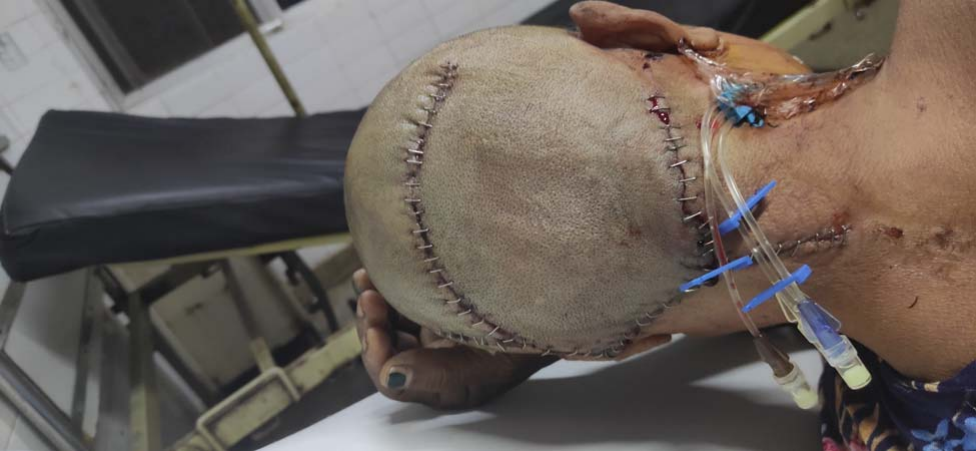
Postoperative state.

**Figure:4:**
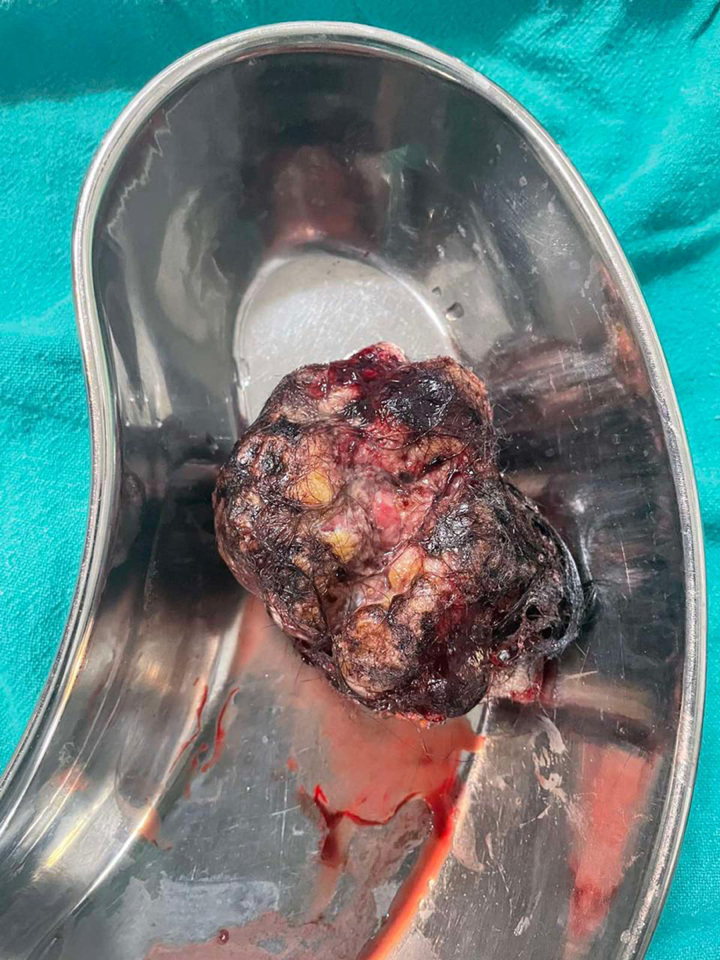
The excised epidermal cyst.

Figure 3 shows the postoperative state of the lesion after excision in which the incised margin approximated with sutures. Figure 4 shows the excised cyst.

## Discussion

Epidermal cysts can occur on various parts of the body and frequently appear on the trunk, face, neck, and scrotum, behind the ears, and in the palmoplantar region. These cysts are mobile unless fibrosis is present. Occasionally, a dark keratin plug can be seen overlying the cyst cavity. These cysts are usually asymptomatic until they become infected or enlarged to damage the adjacent anatomical structures.

Multiple case reports show rare descriptions of giant epidermal cysts of the face, neck, scalp, presternal area, buttocks, penis, and forefoot^1–6^. Baek *et al*.^1^ described a huge epidermal cyst of 15 cm diameter over the right side of the face extending up to the temporal area. Polychronidis *et al*.^2^ reported an epidermoid cyst on the left buttock measuring 28×14×12 cm. Antoszweski *et al*.^3^ described an epidermal cyst of 6×7 cm in size in the occipital region present since 3 years of age and gradually progressed in size. Gasmi *et al*.^4^ described a presternal cyst of diameter 7 cm, which was painless, round, and mobile and present in the child of 3 years since the neonatal period following trauma. Ozawa *et al*.^5^ described a giant epidermal cyst extending from the sole to the dorsum of the foot by penetrating the interosseous muscles of size 8 cm×6 cm×2 cm. Kim *et al*.^6^ described an epidermal cyst on the right posterior neck of size 8 cm in diameter adjacent to posterior neck muscles. Prat Acín and Galeano^7^ described a giant occipital intradiploic epidermal cyst of size 6 cm in diameter which was associated with iatrogenic rupture. Cho *et al*.^8^ described epidermal cyst at the left frontoparietal scalp of size 5 cm diameter with intradiploic involvement with perforation of dura and brain parenchyma with communication to the intracranial structures. Duan *et al*.^9^ described intradiploic epidermal cyst of size 6 cm in diameter located in the posterior cranial fossa with intrusion of occipital bone.

## Conclusion

Via this case learning, it can be stated that epidermal cysts are a slow-growing benign tumor, that can cause diagnostic difficulties, especially when located in the scalp area. In addition to that, when cranial bones and intracranial structures are affected by the cyst, they can even lead to complications and interventional difficulties. Imaging techniques like computed tomography, magnetic resonance imaging or ultrasonography are crucial to determine if there is communication between the cyst and intracranial structures.

## Methods

The work has been reported in line with the SCARE Criteria^10^.

## Ethical approval

The ethics approval is not required for our Case Report. The ethical approval has been obtained from the Institutional Review Committee of BPKIHS.

## Consent

Written informed consent was obtained from the patient for the publication of this case report and accompanying images. A copy of the written consent is available for review by the Editor-in-Chief of this journal on request.

## Source of funding

The study had no funding involved.

## Author contribution

A.D.: conceptualization, data curation, investigation, methodology, validation, and supervision; R.K.G.: conceptualization and validation; J.J.M.: investigation, methodology, and validation; D.K.: writing – original draft, review, and editing, data curation, investigation, methodology, and visualization; A.B.: writing – original draft, review, and editing, data curation, investigation, methodology, and visualization.

## Conflicts of interest disclosure

The authors declare no conflicts of interest.

## Guarantor

All the authors are guarantors of the Case Report.

## Provenance and peer review

Not commissioned, externally peer-reviewed.
